# Case report: A new pathogenic variant of LRBA deficiency with a complex phenotype and Rosai-Dorfman disease

**DOI:** 10.3389/fimmu.2022.944810

**Published:** 2022-12-09

**Authors:** Francesco Fabozzi, Rita De Vito, Stefania Gaspari, Fabrizio Leone, Maurizio Delvecchio, Emanuele Agolini, Federica Galaverna, Angela Mastronuzzi, Daria Pagliara, Maria Antonietta De Ioris

**Affiliations:** ^1^ Department of Hematology/Oncology, Cell and Gene Therapy, Bambino Gesù Children’s Hospital, Istituto di Ricovero e Cura a Carattere Scientifico (IRCCS), Rome, Italy; ^2^ Department of Pediatrics, University of Tor Vergata, Rome, Italy; ^3^ Department of Pathology, Bambino Gesù Children’s Hospital, Istituto di Ricovero e Cura a Carattere Scientifico (IRCCS), Rome, Italy; ^4^ Department of Maternal Infantile and Urological Sciences, Sapienza University of Rome, Rome, Italy; ^5^ Department of Pediatrics, Policlinico Giovanni XXIII, Bari, Italy; ^6^ Laboratory of Medical Genetics, Bambino Gesù Children’s Hospital, Istituto di Ricovero e Cura a Carattere Scientifico (IRCCS), Rome, Italy

**Keywords:** LRBA deficiency, Rosai-Dorfman’s disease, neonatal diabetes, primary immunodeficiencies, autoimmunity

## Abstract

We reported a new pathogenic variant of LRBA deficiency with a complex phenotype—neonatal diabetes, very early-onset inflammatory bowel disease, and polyarthritis—who presented with lymph node enlargement. A case of Rosai-Dorfman’s disease (RDD) was confirmed. The occurrence of an RDD lesion in LRBA-deficiency has never been reported so far.

## Introduction

Lipopolysaccharide-responsive vesicle trafficking, beige-like anchor protein (LRBA) is one of nine known mammalian BEACH domain-containing proteins (BDCP) (1). Individuals homozygous for LRBA variants segregated with the disease usually show autoimmune manifestations, recurrent infections, and hypogammaglobulinemia. Immunological findings include impaired B-cell development (increased numbers of CD21^low^ B cells and decreased class-switched and marginal-zone B cells), impaired plasma cell formation impairment, defective *in vitro* B-cell activation, low proliferative responses, low immunoglobulin secretion, and a deficiency of CD4 T-regulatory (Treg) cells (2–4). LRBA regulates intracellular trafficking of Cytotoxic T-Lymphocyte Antigen 4 (CTLA-4), a negative immune regulator that is constitutively expressed on Treg cells and plays a crucial role in immune peripheral tolerance (5–8). Immune dysregulation, lymphoid organ enlargement, and hypogammaglobulinemia are the main clinical features observed in LRBA-deficient patients, often mimicking autoimmune lymphoproliferative syndrome (ALPS) (9, 10). Recurring infections, especially in the lung and gastrointestinal tract, usually occur in affected patients, while immune-mediated diabetes, enteropathy, and hypogammaglobulinemia are reported in 50% of cases (1). Recent findings strongly support hematopoietic stem cell transplantation (HSCT) in patients with severe presentations of LRBA deficiency (11).

RDD is an uncommon histiocytic disorder originally described in 1969 as “sinus histiocytosis with massive lymphadenopathy” (12). The classic presentation is bilateral cervical lymphadenopathy affecting children or young adults, but extra-nodal disease can affect a significant proportion of patients (13); RDD can occur as an isolated disorder or in association with autoimmune, malignant, or hereditary diseases (14). In the new histiocytosis classification by the Histiocyte Society, RDD is part of the “R group,” which includes sporadic RDD (classical, extra-nodal, or associated with neoplasia or immune disease) and familial RDD (15). This last one comprises inherited conditions predisposing to RDD or RDD-like lesions, such as H syndrome and ALPS.

We herein report a case of LRBA deficiency presenting as neonatal diabetes, very early inflammatory bowel disease (IBD), polyarthritis, and inguinal lymphadenopathy. The histological aspect of this unique reactive lymph node showed aspects resembling RDD.

## Case report

A 6-month-old baby presented with type 1 diabetes mellitus, requiring insulin replacement therapy. Given the very early onset, next-generation sequencing (NGS) for monogenetic diabetes was performed, revealing two compound heterozygous loss-of-function variants in the LRBA gene (NM_001199282), c. 1963C> T (p.Arg655Ter) and c.2999_3000dup (p.Ser1000TyrfsTer2), inherited from the father and the mother, respectively. While the variant p.Arg655Ter has already been described in association with LRBA deficiency, the variant p.Ser1000TyrfsTer2 (c. 2999_30) was never reported and is not present in the Human Gene Mutation Database (HGMD) or in the Genome Aggregation Database (gnomAD). Both variants can be classified as pathogenic according to the American College of Medical Genetics and Genomics criteria. By the age of 7 months, he had developed septicemia from *Staphylococcus aureus* and *Klebsiella* spp., treated with parenteral antibiotics; he presented a direct positive Coombs test in the absence of hemolysis and neutropenia, but the anti-neutrophil antibody assay was negative. Lymphocyte and immunoglobulin counts were within the normal range. Afterwards, the child developed non-infectious diarrhea and a failure to thrive; serological testing for celiac disease and specific IgE for cow’s milk were negative. Esophagogastroduodenoscopy (EGDS) was performed, which reported atrophy of the duodenal mucosa. Gastrointestinal biopsy revealed duodenal mucosa with absence of villi and presence of dense inflammatory lymphoplasmacellular infiltrate in the lamina propria with aspects of cryptitis (CD3: 12/100). He also performed a colonoscopy, which showed preserved machine architecture and machine secretion from the gland crypts. Those findings suggested an autoimmune poly-glandular syndrome, an IPEX-like syndrome; however, in view of the genetic examination carried out previously, no new testing was necessary. Concurrent with the onset of diarrhea, he presented polyarthritis involving both the last finger of his left hand and lower limbs (on the right: II and V ray; on the left: ankle swelling). Treatment with mesalazine, abatacept, and intra-articular steroid injections was started, with improvements in both enteropathy and arthritis.

**Figure 1:**
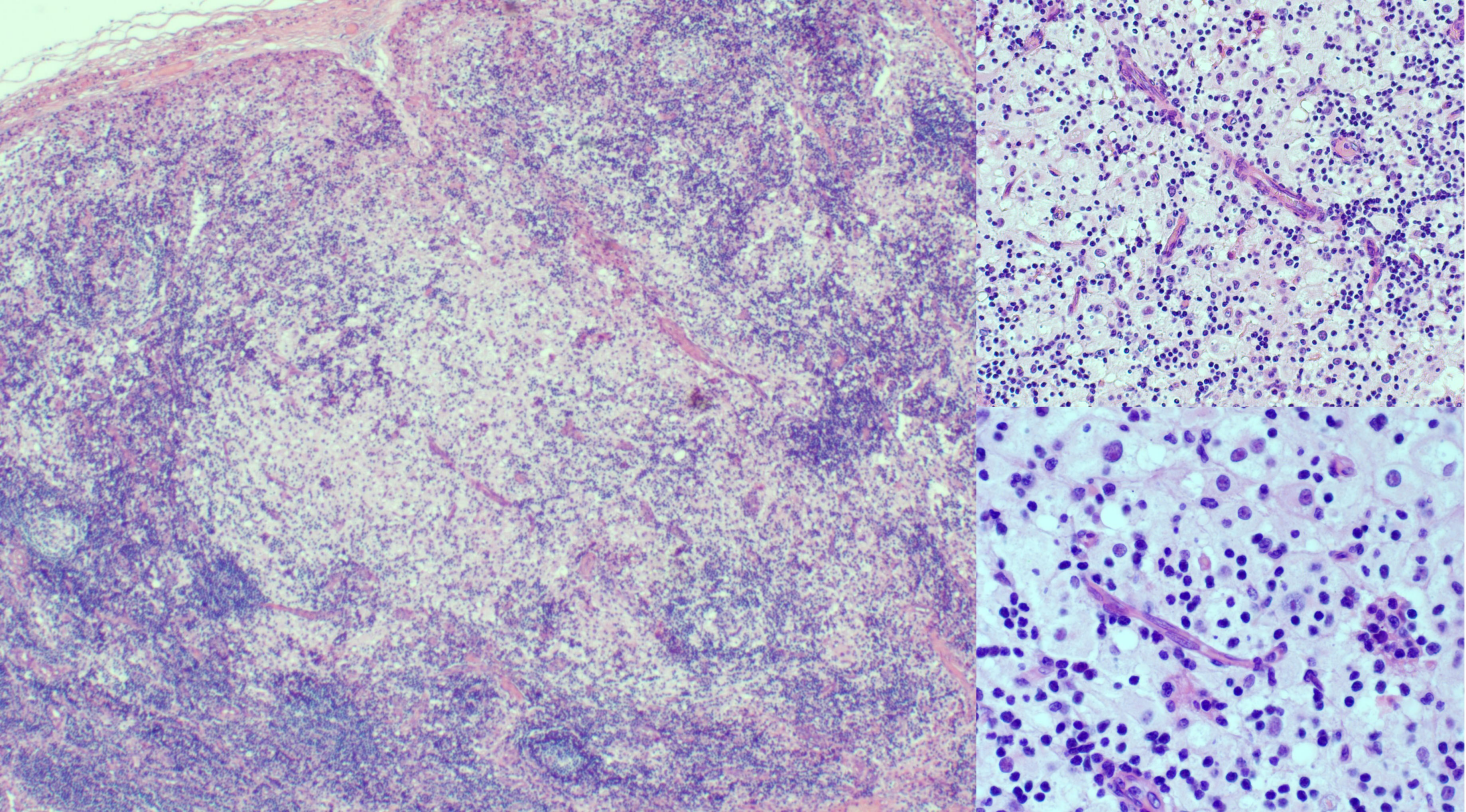
At hematoxylin and eosin (H&E), inguinal lymph node presented an almost normal architecture with a marked expansion of the sinuses occupied by a histiocytic element proliferation. The histiocytic elements appeared voluminous, with a round nucleus and vesicular chromatin, evident but not prominent nucleoli, and abundant eosinophilic cytoplasm. The follicles show aspects of activation and involution and appear depleted in lymphocytes.

**Figure 2:**
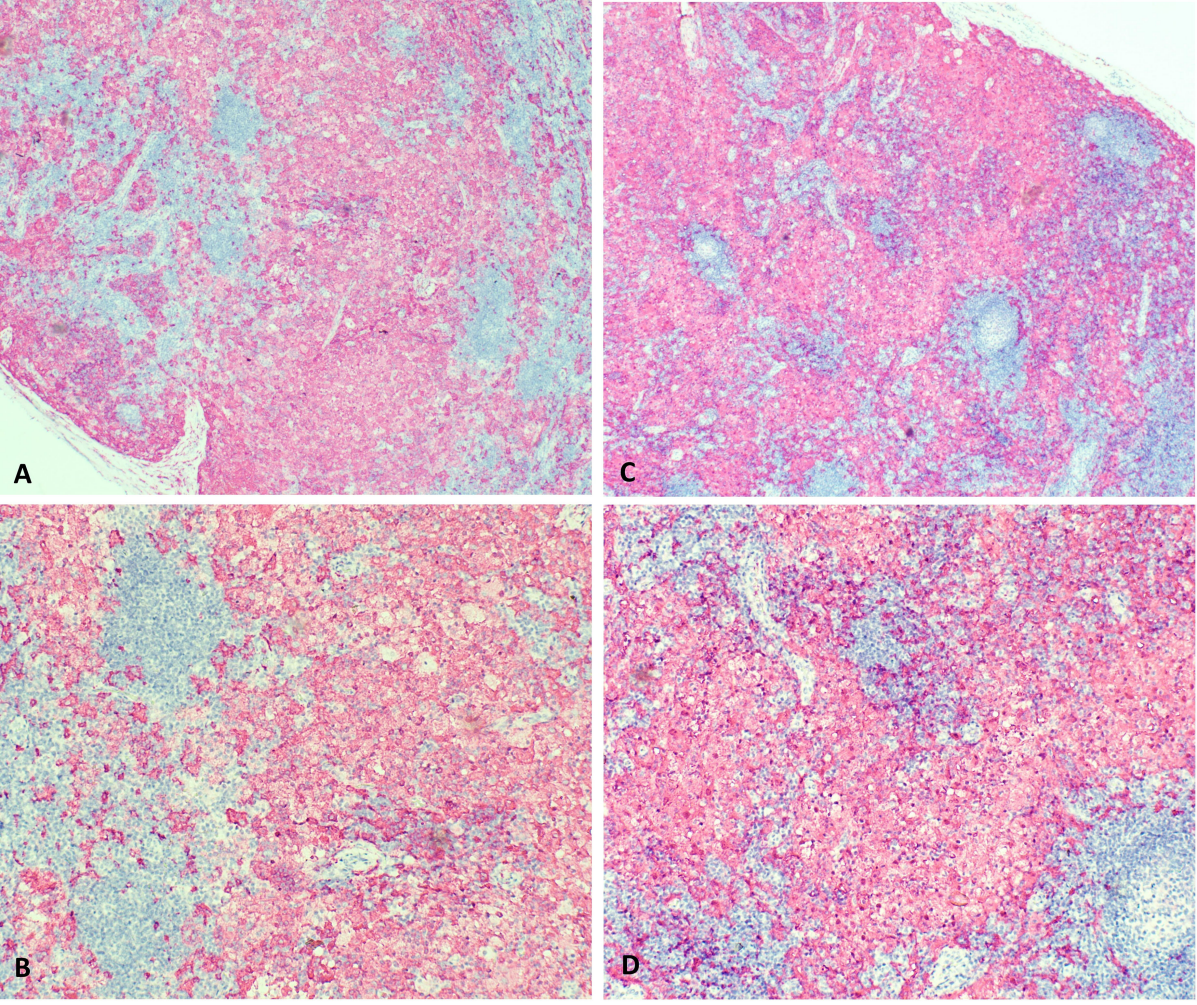
On immunohistochemical staining, the histiocytic cells are S100+, CD31+, CD68+, CD163+, CD14+, Fascine+, CD34 nonspecific, CD1a- and Langherine-. (**A, C**: 5x magnification; **B, D**: 10 x magnification).

**Figure 3:**
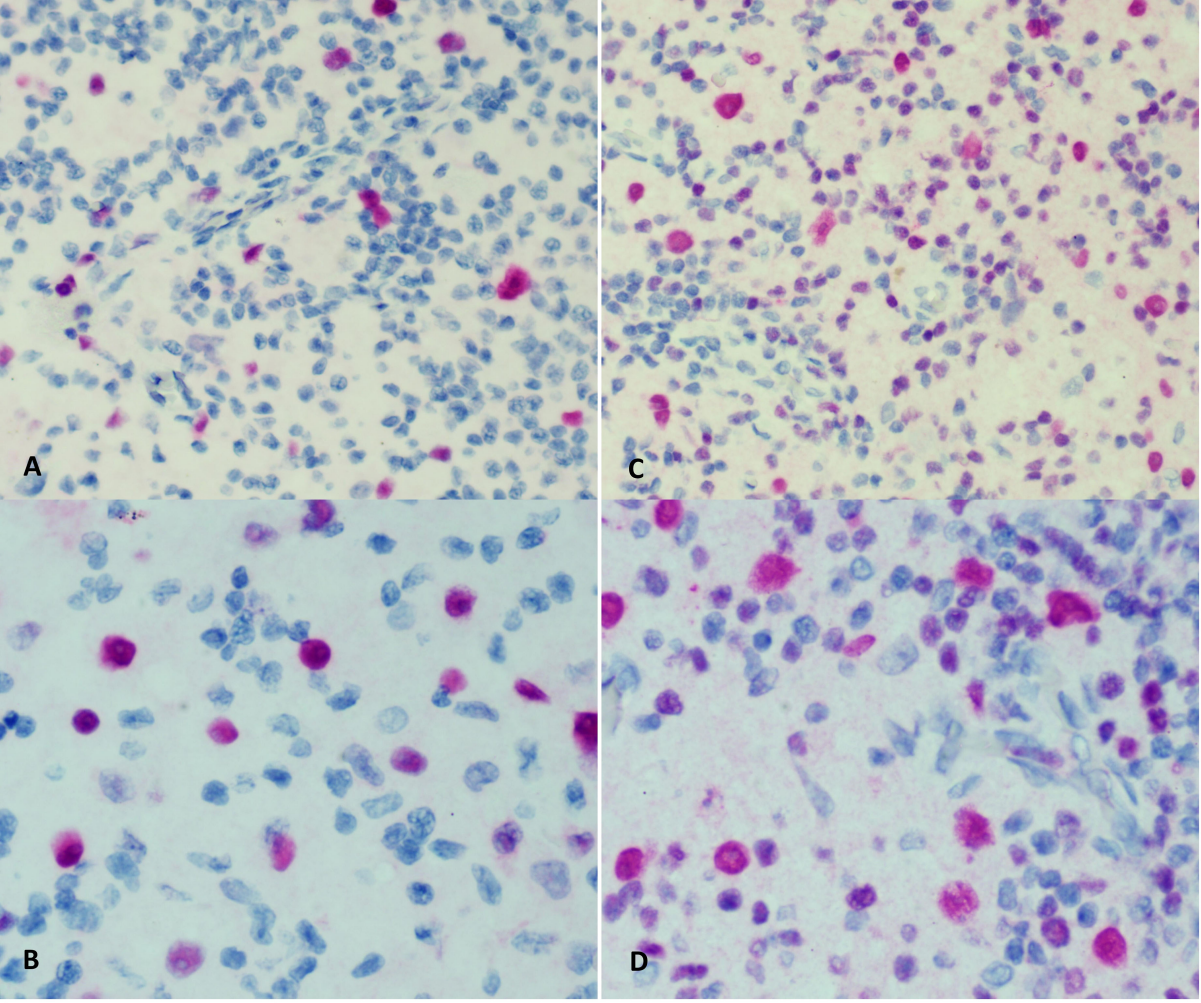
Immunohistochemical staining for cyclin D1 **(A, B)** and OCT2 **(C, D)**.

At 9 months of age, the patient developed unilateral right inguinal lymphadenopathy with positive FDG-PET in the absence of further symptoms. Surgery was planned, and on histological examination, the excised lymph node revealed the morphological picture of RDD. The lymph node presented a distortion of normal architecture with a marked expansion of the sinuses occupied by proliferating histiocytic cells. The histiocytic elements appeared voluminous, with a rounded nucleus, open, vesicular chromatin, evident but not prominent nucleoli, and abundant eosinophilic clear cytoplasm frequently containing vacuoles with intact inflammatory cells (emperipolesis) (**Figure 1**). We observed focal nodular paracortical expansion. In immunohistochemical staining, the histiocytic cells are S100+, CD31+, CD68+, CD163+, CD14+, Fascine+, CD31+, CD1a−, and Langherine− (**Figure 2**). According to Ravidrant et al. (16), OCT2 and cyclin D1 stain was performed and the lesional cells resulted positive for both antibodies (**Figure 3**).

Flow cytometry performed on peripheral blood showed a normal lymphocyte count (3.06 × 10^9^/L), with no T-cell abnormalities nor double-negative T-cell expansion.

## Discussion

RDD is a rare non-Langerhans cell histiocytosis, with a prevalence of 1:200,000 (17). Activated histiocytes (typically S100+, CD68+, and CD1a−, with a variable frequency of emperipolesis) were detected within affected tissues (14). A broad spectrum of clinical presentations characterizes RDD, ranging from classic bilateral cervical lymphadenopathy to extra-nodal forms that occur in 43% of cases (18). Indeed, RDD may occur in an isolated form or in association with neoplastic or autoimmune diseases. As highlighted by the new histiocytosis classification, a fraction of cases is associated with hereditary syndromes, with H syndrome in ALPS being the most important (15). ALPS is a primary immune regulatory disorder due to an apoptotic defect in the Fas-FasL pathway. This results in the expansion and accumulation of autoreactive (double-negative) T cells, leading to chronic lymphoproliferation, autoimmunity, and an increased risk of lymphoma (10). In a study by Maric et al., 18 of 44 patients affected by ALPS were found to have RDD-like lymphadenopathy. Furthermore, LRBA deficiency leads to impaired trafficking of CTLA-4 on the Treg cells’ surface, causing disruption of immune homeostasis and leading to autoimmune disorders, organomegaly, and hypogammaglobulinemia. Clinical pictures overlap with ALPS. The pathology features lead to a RDD diagnosis.

LRBA deficiency is a disease that is not yet well understood. Variable phenotypes can complicate diagnosis and lead to delays. In this case, considering the early onset of type 1 diabetes mellitus, next-generation sequencing (NGS) was performed, leading to the diagnosis. The clinical picture comprises polyarthritis, enteropathy, and recurrent infections. A single lymph node enlargement with RDD-like pathological characteristics was found. Despite the well-known association between RDD and a primary immune regulatory disorder such as ALPS, it has never been reported in LRBA deficiency.

We reported a new pathogenic variant of LRBA deficiency with a complex phenotype—neonatal diabetes, very early-onset inflammatory bowel disease, and polyarthritis—who presented with lymph node enlargement with an RDD diagnosis. This is the first report on the occurrence of an RDD lesion in LRBA deficiency.

## Data availability statement

The original contributions presented in the study are included in the article/supplementary material. Further inquiries can be directed to the corresponding author.

## Ethics statement

Written informed consent was obtained from the minor(s)’ legal guardian/next of kin for the publication of any potentially identifiable images or data included in this article.

## Author contributions

MADI and AM conceived of the study. MAD, FF, and FL drafted the manuscript. EA and RDV performed the data analysis. All authors listed have made a substantial, direct, and intellectual contribution to the work and approved it for publication.
